# Altered prefrontal beta oscillatory activity during removal of information from working memory in obsessive-compulsive disorder

**DOI:** 10.1186/s12888-023-05149-1

**Published:** 2023-09-04

**Authors:** Young Jun Boo, Do-Won Kim, Jin Young Park, Bong Soo Kim, Jin Woo Chang, Jee In Kang, Se Joo Kim

**Affiliations:** 1 Department of Psychiatry, Graduate School, Yonsei University College of Medicine, Seoul, Republic of Korea; 2 Department of Biomedical Engineering, College of Engineering Sciences, Chonnam National University, 50 Daehak-ro, Yeosu, Republic of Korea; 3 School of Healthcare and Biomedical Engineering, College of Engineering Sciences, Chonnam National University, 50 Daehak-ro, Yeosu, Republic of Korea; 4 Department of Psychiatry, Institute of Behavioral Science in Medicine, Yonsei University College of Medicine, 50 Yonsei-ro, Seodaemun-gu, Seoul, Republic of Korea; 5 Department of Psychiatry, Yongin Severance Hospital, Yonsei University College of Medicine, Yongin, Republic of Korea; 6 Department of Neurosurgery, Brain Research Institute, Yonsei University College of Medicine, Seoul, Republic of Korea

**Keywords:** Obsessive-compulsive disorder, Working memory, Removal of information, Delayed matching-to-sample task, Beta oscillations, Magnetoencephalography

## Abstract

**Background:**

Obsessive-compulsive disorder (OCD) is related to working memory impairment. Since patients with OCD have difficulty controlling their obsessive thoughts, removal of irrelevant information might be important in the pathophysiology of OCD. However, little is known about brain activity during the removal of information from working memory in patients with OCD. Our goal was to explore potential deficits in inhibitory function related to working memory processes in patients with OCD.

**Methods:**

Sixteen OCD patients and 20 healthy controls (HCs) were recruited. We compared in prefrontal alpha and beta band activity derived from magnetoencephalography (MEG) between patients with OCD and HCs during multiple phases of information processing associated with working memory, especially in post-trial period of the visuospatial working memory task (the delayed matching-to‐sample task), which is presumed to be related to the information removal process of working memory.

**Results:**

Prefrontal post-trial beta power change (presumed to occur at high levels during the post-trial period) exhibited significant reductions in patients with OCD compared to HCs. In addition, the post-trial beta power change was negatively correlated with Obsessive-Compulsive Inventory–Revised total scores in patients with OCD.

**Conclusions:**

These findings suggest that impairment in the removal of information from working memory might be a key mechanism underlying the inability of OCD patients to rid themselves of their obsessions.

## Background

Obsessive-compulsive disorder (OCD) is characterized by recurrent intrusive thoughts that are hard to ignore or suppress (obsessions) and repetitive behaviors employed to resist or eliminate these obsessive thoughts (compulsions) [1]. A fundamental deficit in inhibitory control, which is critically dependent on the prefrontal cortex, has been implicated in these clinical features of OCD [2]. Substantial evidence from neuroimaging and neuropsychological studies suggests that functional abnormalities in fronto-striatal brain circuits [3–5] and that impairments in neurocognitive functions subserved by the fronto-striatal circuits are key psychopathological mechanisms underlying OCD [6, 7].

Despite mixed and inconsistent findings across studies and neuropsychological domains, accumulating evidence suggests the presence of neurocognitive deficits in OCD patients. A meta-analysis highlighted reduced performance in all domains of attention, executive function, memory, processing speed, visuospatial abilities, and working memory in people with OCD, compared to healthy controls (HCs), with various effect sizes of the between-group differences across domains: a large effect size was found for the nonverbal memory domain, with medium effect sizes for attention, executive functions, and processing speed and small effect sizes for working memory [8]. Another meta-analysis indicated broad-ranging neuropsychological deficits, except in attentional ability, in people with OCD compared to HCs: the largest effect size was found for visuospatial memory, and small-to-medium effect sizes were found for executive function, processing speed, verbal fluency, and verbal memory [9]. A meta-analysis on executive function in OCD patients showed that people with OCD exhibited impaired performance in most domains of executive functions with small-to-medium effect sizes [6]. Despite inconsistent evidence, visuospatial memory impairment has been relatively consistently reported in OCD patients and is proposed to be secondary to impaired executive control, such as strategy failures [8]. However, the precise nature of cognitive deficits, including visuospatial memory impairments, in OCD, is still poorly understood. Decades of research into neural activities associated with these neurocognitive deficits has provided evidence supporting impaired frontal inhibition, such as reduced task-related frontal alpha activity, in OCD patients, but the results are highly inconsistent and heterogeneous across studies [10].

Working memory, the ability to actively hold, manipulate, and update information in one’s mind over a short period, is one of the main executive functions supported by the prefrontal cortex [11, 12]. Due to its limited capacity, the removal of irrelevant information is essential to making room for new information and maintaining prioritized content [13, 14]. Recent behavioral and neurophysiological evidence supports the existence of an information removal process in working memory [15]. A recent experimental study using a visuospatial working memory task revealed that working memory is successfully updated by reallocating resources from previous obsolete memories to new information [16]. In addition, brain activity patterns involved in removing information from working memory were reported [14]. Research on removing and updating working memory is important to understanding the symptoms and etiology of neuropsychiatric disorders related to disengaging from information in working memory. To date, the removal of no-longer-relevant information from working memory has received little attention in clinical research.

Deficits in the removal of no-longer-relevant information from working memory may play a critical role in OCD, as OCD patients experience difficulties in controlling unwanted intrusive thoughts. To understand the characteristics and the underpinning neural activity in OCD, we recorded prefrontal activity using magnetoencephalography (MEG), which has high spatial and temporal resolution [17], during a visuospatial delayed matching-to-sample task (DMST). The DMST has been commonly used to examine temporal properties and the neural basis of working memory, and the prefrontal cortex has been shown to be a key component for attending to an appropriate stimulus and inhibiting any irrelevant information during the task [18]. The DMST includes sample/encoding, delay/retention, and choice/retrieval phases in each trial and requires top-down inhibitory control of irrelevant information to clear the memory buffer after each single trial [19]. In the post-trial period, an animal experiment on time-varying signals in working memory delay activity showed increased beta bursts in the post-trial epoch when encoded information was no longer needed after the behavioral response [20]. While both alpha and beta oscillations are known to be linked to inhibitory processes in working memory, prefrontal beta oscillations occurring at high levels at the end of the trial may be related to the removal of irrelevant information, a mechanism for working memory reallocation [20, 21]. The excellent temporal resolution of MEG enables the measurement of cortical activity in different frequency bands within the same time frame over the distinct consecutive phases of a working memory task [17, 19]. Using MEG recordings during the DMST, Ciesielski et al. showed enhanced activation within the prefrontal network [22] and a reduction in frontal alpha modulation in OCD patients, particularly during the retention and retrieval phases of the DMST containing distractors, suggesting impaired inhibition and abnormal allocation of cognitive resources during working memory maintenance in OCD patients [23]. However, they did not analyze the post-trial period. To our knowledge, no neurophysiological studies have examined the removal of information from working memory in OCD patients.

The present study aimed to examine prefrontal alpha and beta band activity from MEG signals during multiple phases of information processing associated with working memory using a case-control design and enrolling OCD patients. In particular, we analyzed data from the post-trial period, which could represent the removal of information from working memory, in addition to three distinct phases (encoding, retention, and retrieval) in a visuospatial DMST. We expected to observe task-phase specific alterations of prefrontal alpha and beta oscillations engaging the visuospatial working memory in OCD patients. In particular, we hypothesized that prefrontal beta oscillations (presumed to occur at high levels during the post-trial period) would be reduced in patients with OCD compared to HCs.

## Materials and methods

### Participants

The present study included 16 patients with OCD (mean age = 23.56 years, SD = 2.37 years, 16 men) and 20 HCs (mean age = 22.50 years, SD = 2.40 years, 20 men). Only young male participants aged between 20 and 29 years were included to control for any effects due to sex and age. In addition, for inclusion in the study, subjects had to have an intelligence quotient higher than 85 based on scores obtained with the short form [24] of the Korean version of the Wechsler Adult Intelligence Scale–IV [25]. All participants were right-handed. Participants were assessed by a trained psychiatrist using the Structured Clinical Interview for the Diagnostic and Statistical Manual of Mental Disorders, fourth edition (DSM-IV) [26], to evaluate the presence of current or past psychiatric disorders. Demographic and clinical information was also assessed. The patients with OCD were recruited from an outpatient clinic specializing in OCD at Severance Hospital of Yonsei University Health System (Seoul, South Korea), a tertiary hospital. For inclusion in the OCD group, individuals had to have a primary diagnosis of OCD and no lifetime history of other anxiety disorders, schizophrenia spectrum disorders, bipolar disorder, or substance dependence, as defined by the DSM-IV. In addition, those with a history of head trauma and severe organic or neurologic disorders were excluded. All patients with OCD received pharmacotherapy and brief cognitive-behavioral therapy in routine outpatient care. For inclusion in the control group, healthy recruited through internet advertisements had to have no lifetime history of any psychiatric disorders according to the DSM-IV diagnostic criteria.

All subjects included in the study provided written informed consent prior to participation. The present study protocol was approved by the Institutional Review Board of Yonsei University (IRB number: 1-2011-0088), and the study was conducted in accordance with the Declaration of Helsinki.

### Measurements

#### Montgomery-Åsberg Depression Rating Scale (MADRS)

We used the Korean validated version of the Montgomery-Åsberg Depression Rating Scale (MADRS) to assess the severity of depressive symptoms in all of the participants [27, 28]. The MADRS consists of 10 clinician-administered diagnostic items, including various depressive symptoms.

#### Yale-Brown Obsessive compulsive scale (Y-BOCS) and Y-BOCS Symptom Checklist

The Yale-Brown Obsessive Compulsive Scale (Y-BOCS) has been used to evaluate obsessive-compulsive symptoms in patients with OCD [29]. The Y-BOCS is a validated clinician-administered scale that consists of 10 items used to assess the severity of obsessive and compulsive symptoms. In addition, the 58-item Y-BOCS Symptom Checklist was employed to identify the symptom dimensions of OCD. Based on a previous meta-analysis of the dimensional structure of OCD [30], symptom dimensions were classified into four categories: symmetry (symmetry obsessions and repeating, ordering, and counting compulsions), forbidden thoughts (aggressive, sexual, religious, and somatic obsessions and checking compulsions), cleaning (contamination obsessions and cleaning compulsions), and hoarding (hoarding obsessions and compulsions). The presence of a symptom dimension was determined according to a lifetime history of one or more symptoms in the respective category [Table 1].

**Table 1:** Demographic and clinical characteristics of patients with OCD and healthy controls

	OCD (n = 16)	HC (n = 20)	t (z)	p
Age (years)	23.56 ± 2.37	22.50 ± 2.40	1.330^a^	0.192
MADRS	14.56 ± 8.97	2.95 ± 4.12	4.055^b^	< 0.001***
OCI-R-K	38.50 ± 18.63	16.85 ± 12.09	4.213^a^	< 0.001***
Y-BOCS	17.69 ± 7.85			
Symptom dimension, present, n (%)				
Symmetry	12 (75.0%)			
Forbidden thoughts	12 (75.0%)			
Cleaning	11 (68.8%)			
Hoarding	10 (62.5%)			
Use of SSRI, n (%)	15/16 (93.8%)			
Use of Benzodiazepine, n (%)	8/16 (50.0%)			

OCD, obsessive-compulsive disorder; HC, healthy control; MADRS, Montgomery-Åsberg depression rating scale; OCI-R-K, Korean version of obsessive-compulsive inventory-revised; Y-BOCS, Yale-Brown obsessive compulsive scale; SSRI, selective serotonin reuptake inhibitor

^a^ t of independent sample t-tests, ^b^ z of Mann-Whitney U test

* significant at p < 0.05, ** significant at p < 0.01, *** significant at p < 0.001

#### Korean version of the obsessive-compulsive inventory–revised (OCI-R-K)

We used the Korean version of the Obsessive-Compulsive Inventory–Revised to evaluate obsessive-compulsive symptoms in patients with OCD and HC [31]. This self-report scale includes 18 items to assess distress related to obsessions and compulsions, with higher scores indicating more severe symptoms of OCD.

#### Delayed matching-to-sample task (DMST)

**Fig. 1:**
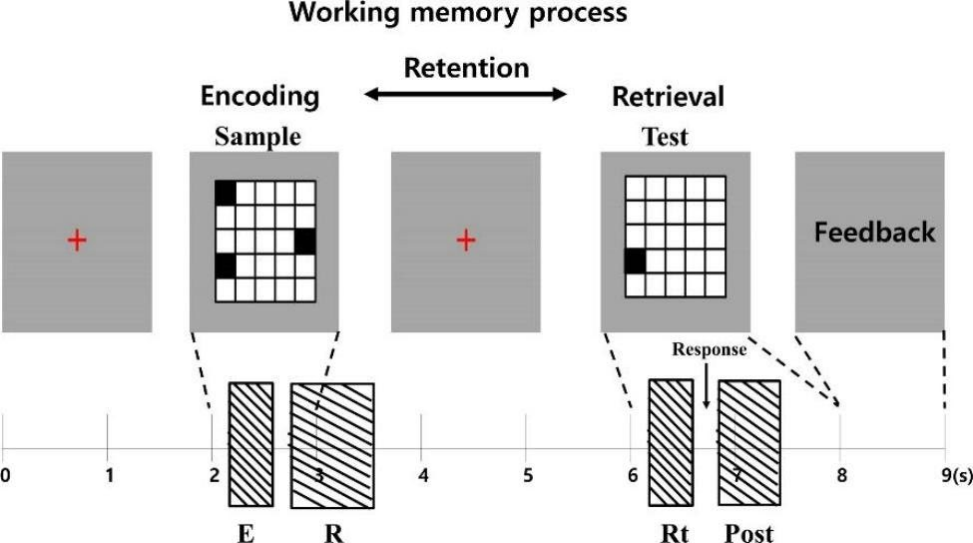
Delayed matching-to-sample task used in the study. The boxes indicate the time window of interest related to each period of working memory processes (E, encoding period; R, retention period; Rt, retrieval period; and Post, post-trial period)

The visuospatial DMST, a working memory task, was selected for this study [Fig. 1]. It is useful for evaluating brain activity during working memory processes since working memory processes, including encoding, retention, and retrieval, can be differentiated in the task [20]. The task involves presenting two sets of objects separately, and subjects are asked to respond whether the later (delayed) presented object matches the previously presented object [32]. As reported in a previous electroencephalography (EEG) study [20], data from the post-trial period of the DMST can indicate removal of no-longer-needed information from working memory after retrieval.

The design of task cues was based on a previous OCD study in which visuospatial working memory was explored using MEG [22, 23]. The task consisted of 120 pseudorandomly presented trials. Each trial started with a small red cross presented for 2,000 ms. Afterward, a 5 × 5 square with 3 black-colored squares was shown for 1,000 ms. This was followed by a 3,000 ms delay. Then, the probe consonant was shown for 2,000 ms. Participants were asked to respond as quickly and accurately as possible and indicate if the probe showed one of the black squares previously shown. Feedback on the answer and the accuracy rate were presented for 1,000 ms at the end of each trial. The participants performed 10 practice trials before the task.

Accuracy and reaction time on the DMST were obtained to evaluate the behavioral performances of the participants.

### MEG data

#### MEG recordings

A 152-channel whole-head MEG device (Korea Institute of Standards and Science; KRISS, Daejeon, Korea) was used to record magnetic fields induced by brain electrical activity. Participants were asked to sit in a magnetically shielded room (Yonsei University Health System, Seoul, Korea). Resting-state recordings were recorded for 3 min each with the eyes closed and with the eyes open. Afterward, all participants were asked to perform the DMST while MEG data were recorded. Magnetic fields were recorded at a sampling rate of 1,000 Hz with a bandpass filter between 0.1 and 100 Hz.

#### MEG data preprocessing

Resting-state recordings were collected only to confirm the reliability of the data and were not used in further analysis. All preprocessing procedures were performed with CURRY 8 (Compumedics, Charlotte, NC, USA) software. First, baseline correction was achieved by subtracting the overall mean of each channel from every point. Gross-movement artifacts and other muscle related noises were identified by a trained specialist and rejected from further analysis. Epochs were extracted in the period of -300 ~ 1,500 ms from each sample cue and testing cue. Only trials with correct answers were included in the analysis.

#### MEG data analysis

##### Spectral analysis

To measure the spectral power, an event-related spectral perturbation (ERSP) analysis was applied to the recorded MEG signals. ERSP was calculated using functions implemented in EEGLAB [33]. Spectral power was calculated using the short-time Fourier transform every 5 ms with a Hanning window size of 250 ms for each trial. No smoothing or filtering process was applied when generating the resultant ERSP maps. The power spectrum of each trial was then normalized against the average power of the partial baseline period (-300 to -100 ms before the sample cue and test cue) to probe for changes in the spectral power values after the onset of the sample cue and test cue. The normalized spectral power was then averaged over the trials, and baseline-normalized ERSP maps for each group of participants were created.

##### Determination of oscillations of interest

Alpha power values for each condition were estimated in a frequency range of 8 to 13 Hz based on previous studies on the encoding, retention, and retrieval processes of working memory in patients with OCD [23, 34]. Beta power values for each condition were estimated in a frequency range of 20 to 35 Hz based on a previous report of the removal of information from working memory in DMST [20].

##### Determination of the time window of interest

The time window in the post-trial period was selected based on the response time of the participants [Table 2]. In a study on brain activity during working memory processes, beta bursts related to the removal of information from working memory started to increase around the participant response time [20]. Other periods besides the post-trial period were analyzed to confirm their consistency with a previous study on brain activity during working memory processes.

Based on the sample cue, the encoding period (300 ~ 600 ms) and retention period (650 ~ 1,500 ms) were established. Based on the test cue, the retrieval period (300 ~ 600 ms) was established. We defined 650 ~ 1,500 ms after the test cue as the post-trial period [Fig. 1].

**Table 2:** Behavioral performance in the delayed matching-to-sample task

	OCD	HC	t(z)	p
Accuracy (%)	91.81 ± 4.69	94.00 ± 3.36	− 1.571^b^	0.200
Reaction time (ms)	765.91 ± 154.66	662.03 ± 95.66	2.351^a^	0.027*

OCD, obsessive-compulsive disorder; HC, healthy control

^a^ t of independent sample t-test, ^b^ z of Mann-Whitney U test

* significant at p < 0.05

### Determination of the regions of interest

**Fig. 2:**
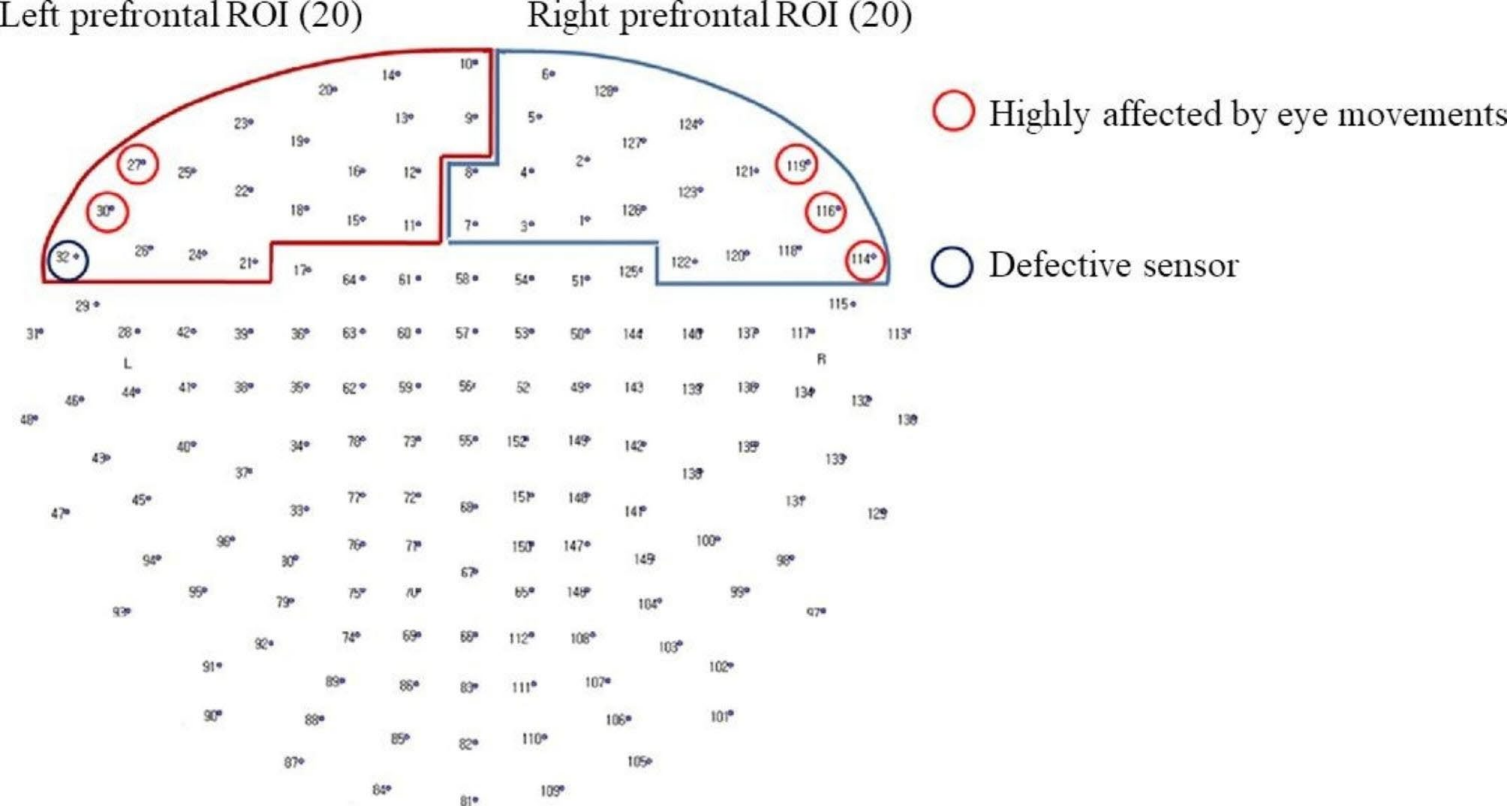
Channel locations and regions of interest (the left/right prefrontal cortex). ROI, region of interest

We included 20 sensors each in the bilateral prefrontal area and set the left/right prefrontal cortex as the region of interest (ROI) [Fig. 2]. Since beta oscillation is related to motor planning extinction in the motor cortex, we attempted to avoid including sensors near the premotor area [21]. Among the sensors in the left prefrontal ROI, 2 were highly affected by eye movements, and 1 was defective. Among the sensors in the right prefrontal ROI, 3 were highly affected by eye movements. These sensors were excluded from the analysis.

### Statistical analysis

All statistical analyses were performed with Statistical Package for the Social Sciences version 26 (SPSS Inc., Chicago, IL, USA). Demographic and clinical data, including age, Y-BOCS scores and MADRS scores, were compared with independent-sample t tests between patients with OCD and HCs. The behavioral performance on the DMST, including the accuracy of the response and the reaction time, were also compared with independent-sample t tests between patients with OCD and HCs. If the demographic, clinical, or behavioral performance variables were not normally distributed, demographic and clinical variables of patients with OCD and HCs were compared using a two-tailed Mann‒Whitney U test. The statistical significance threshold was set at p < 0.05.

For spectral analysis, to compare the mean power changes in decibels (dB) between the two groups, repeated-measures multivariate analysis of covariance (MANCOVA) was performed, with the group (OCD vs. HC) as the between-subject factor, hemisphere (left prefrontal ROI vs. right prefrontal ROI) as the repeated factor, and the MADRS score as a covariate. The MADRS score was included as a covariate in the analyses to control for the effects of depressive symptoms. The MANCOVA included 8 dependent variables reflecting the mean power changes: alpha and beta oscillations during 4 periods (E, encoding; R, retention; Rt, retrieval; and Post, post-trial). Post hoc univariate analysis of covariance (ANCOVA) was run to identify the dependent variables with significant difference between the two groups if the initial MANCOVA results were statistically significant (with a cutoff of 𝛼 = 0.05). For post hoc tests, we adjusted the significance threshold with Bonferroni correction to p < 0.00625 (i.e., 0.05/8 for alpha and beta oscillations in 4 periods). We calculated values of partial eta squared as the effect size, and the values were interpreted according to Cohen’s guidelines of effect sizes: small (η_p_^2^ = 0.01), medium (η_p_^2^ = 0.06), or large (η_p_^2^ = 0.14) [35]. In addition, partial correlation coefficients (*r*), controlling for MADRS scores, were calculated between significant results from the post hoc ANCOVA and obsessive-compulsive symptom severity scores (Y-BOCS and OCI-R-K scores) in patients with OCD. Scatter plots of partial correlation analysis were created.

## Results

### Demographic and behavioral data

The demographic data are presented in Table 1. Age differences between the OCD and HC groups were not statistically significant (OCD = 23.56 vs. HC = 22.50; p = 0.192). The MADRS score was significantly higher in patients with OCD (OCD = 14.56 vs. HC = 2.95; p < 0.001).

The results of behavioral performance on the DMST are presented in Table 2. The accuracy rate was not different between the OCD and HC groups (OCD = 91.81 vs. HC = 94.00; p = 0.128). The reaction time was significantly longer in patients with OCD than in HCs (OCD = 765.91 vs. HC = 662.03; p = 0.027).

### Spectral-power analysis of alpha and beta oscillations in the prefrontal cortex

**Fig. 3:**
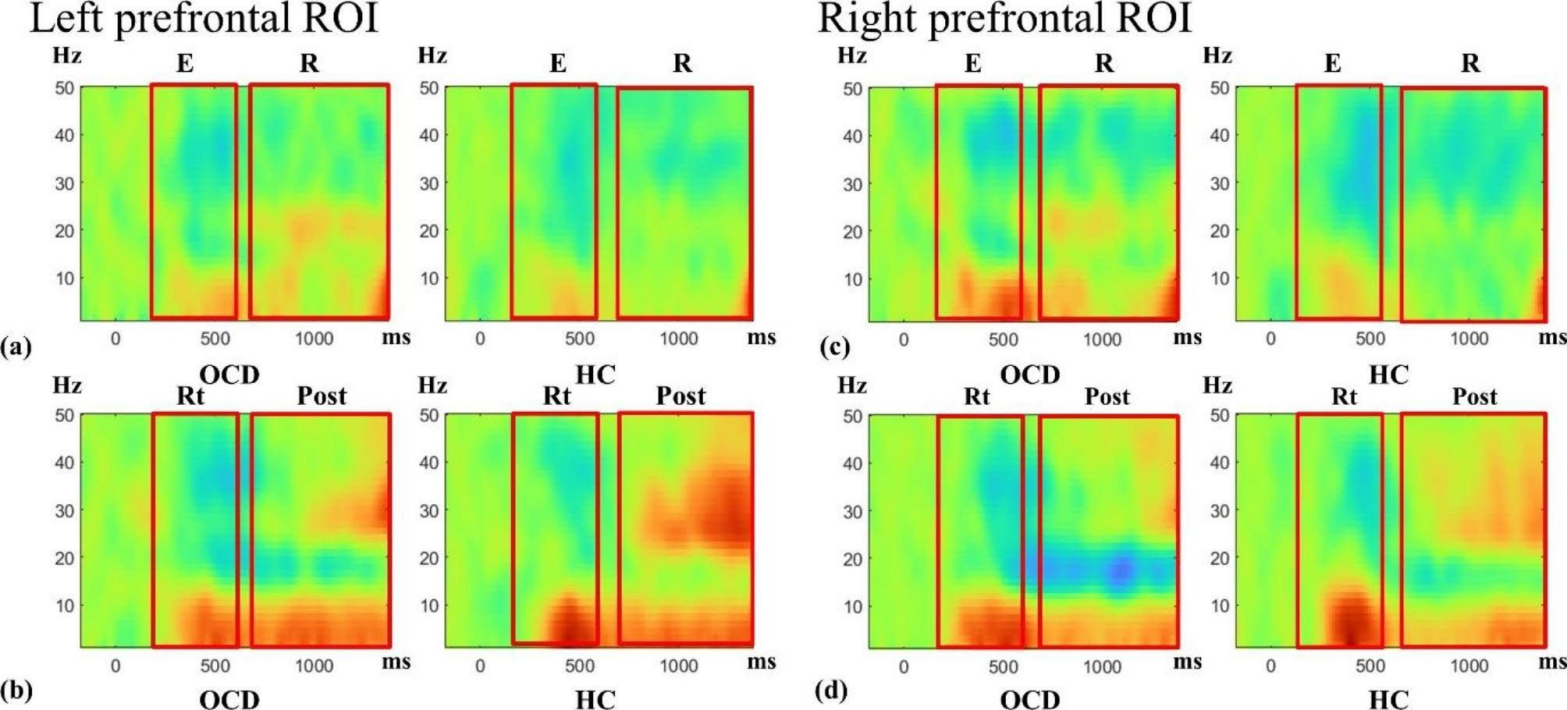
Event-related spectral perturbation (ERSP) map during the delayed matching-to-sample task for prefrontal regions of interest. Red indicates an increase in power, and blue indicates a decrease in power. (**a**) Left prefrontal sample cue, (**b**) left prefrontal test cue, (**c**) right prefrontal sample cue, and (**d**) right prefrontal test cue. Red squares indicate the time windows of interest. ROI, region of interest; OCD, obsessive-compulsive disorder; HC, healthy control; E, encoding period; R, retention period; Rt, retrieval period; and Post, post-trial period

**Fig. 4:**
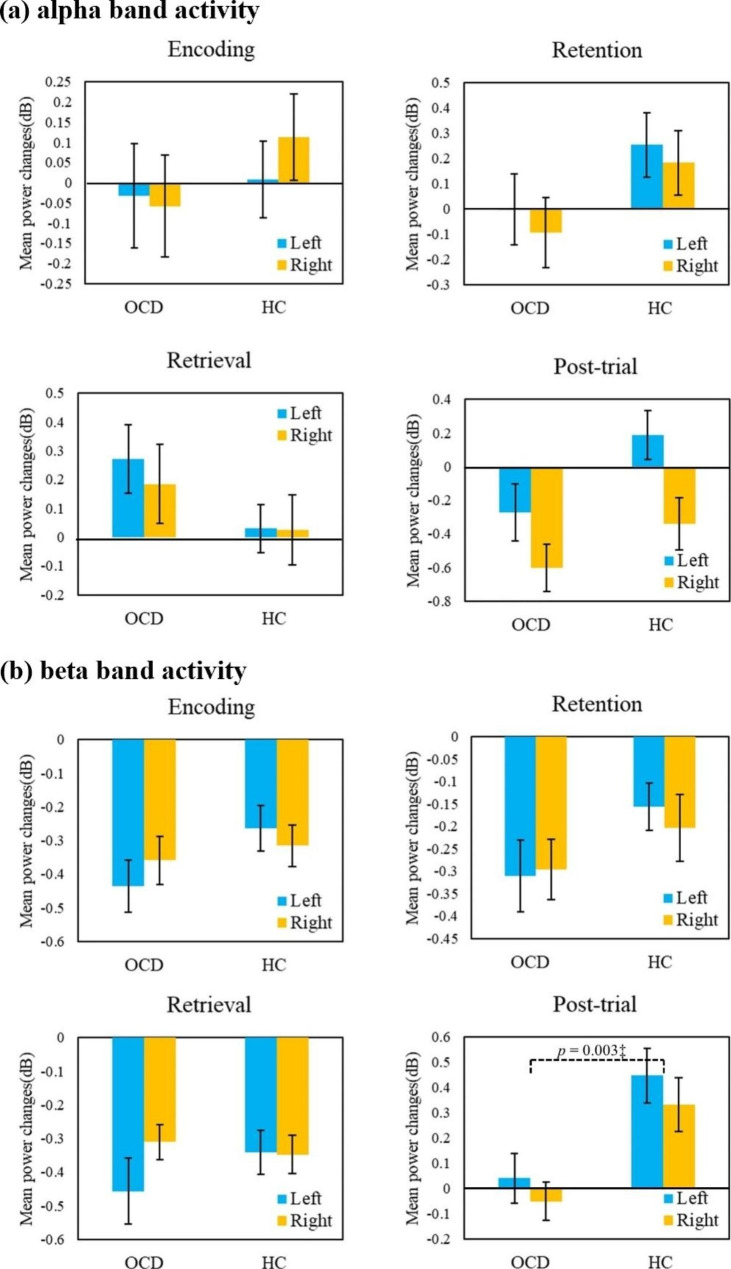
Results from spectral-power analysis of alpha (**a**) and beta (**b**) oscillations in the prefrontal cortex. The graphs represent the mean values of changes in power (dB) in the 16 patients with obsessive-compulsive disorder (OCD) and the 20 control subjects. The error bar shows ± 1 standard error of the mean value. OCD, obsessive-compulsive disorder; HC, healthy control ^‡^ significant at p < 0.00625

The ERSP map during the delayed matching-to-sample task is shown in Fig. 3. The repeated-measures MANCOVA with group (OCD, HC) as the between-subject factor, hemisphere (left, right) as the repeated factor, and alpha and beta oscillations during 4 task periods as the dependent variables showed a significant main effect of group (F_8,26_ = 2.509; p = 0.036; η_p_^2^ = 0.463). There was no significant main effect of hemisphere (F_8,26_ = 2.309; p = 0.051). No significant effects of the interactions between group and hemisphere (F_8,26_ = 0.797; p = 0.611) or between the MADRS score and hemisphere (F_8,26_ = 0.222; p = 0.984) were found. Post hoc tests were run to delineate the nature of the significant finding of the between-subject factor diagnosis. The values acquired from the post hoc tests are presented in Table 3. In the follow-up analyses, for alpha oscillations, no significant between-group differences were found during the encoding (F_1_ = 0.299; p = 0.588), retention (F_1_ = 0.846; p = 0.364), retrieval (F_1_ = 4.717; p = 0.037), or post-trial periods (F_1_ = 1.149; p = 0.292) [Fig. 4a]. For the retrieval phase, the alpha power change was higher in patients with OCD than in HCs (p = 0.037) at the nominal level, but the between-group difference did not reach the significance threshold with Bonferroni correction of p < 0.00625. On the other hand, for beta oscillations, patients with OCD showed a significant reduction in the increase in beta band activity during the post-trial period, with a large effect size (F_1_ = 10.178; p = 0.003; η_p_^2^ = 0.236). No significant between-group differences in the changes in beta band activity were observed during other phases of encoding (F_1_ = 0.378; p = 0.543), retention (F_1_ = 0.856; p = 0.359), or retrieval (F_1_ = 0.147; p = 0.704) [Fig. 4b].

**Table 3:** Group differences in prefrontal alpha and beta oscillations during a visuospatial DMST †

Oscillation	Periods	OCD (n = 16)		HC (n = 20)		F	*p*	η_p_^2^
		Left	Right	Left	Right			
Alpha	Encoding	-0.032 ± 0.520	-0.058 ± 0.508	0.008 ± 0.425	0.113 ± 0.481	0.299	0.588	0.009
	Retention	-0.002 ± 0.561	-0.093 ± 0.555	0.254 ± 0.573	0.182 ± 0.574	0.846	0.364	0.025
	Retrieval	0.272 ± 0.472	0.184 ± 0.549	0.030 ± 0.369	0.025 ± 0.541	4.717	0.037	0.125
	Post-trial	-0.274 ± 0.677	-0.602 ± 0.564	0.188 ± 0.656	-0.340 ± 0.701	1.149	0.292	0.034
Beta	Encoding	-0.437 ± 0.311	-0.359 ± 0.287	-0.264 ± 0.300	-0.315 ± 0.274	0.378	0.543	0.011
	Retention	-0.311 ± 0.322	-0.296 ± 0.271	-0.156 ± 0.236	-0.204 ± 0.333	0.867	0.359	0.026
	Retrieval	-0.457 ± 0.391	-0.311 ± 0.209	-0.341 ± 0.293	-0.349 ± 0.256	0.147	0.704	0.004
	Post-trial	0.041 ± 0.397	-0.051 ± 0.302	0.447 ± 0.486	0.332 ± 0.477	10.178	0.003*	0.236

Values represent means ± standard deviations

DMST, delayed matching-to‐sample task; OCD, obsessive-compulsive disorder; HC, healthy control

† This post hoc test is based on a previous repeated-measures multivariate analysis of covariance; * significant at p < 0.00625

### Partial correlation between prefrontal beta power change during the post-trial period and obsessive-compulsive symptom severity score

**Fig. 5:**
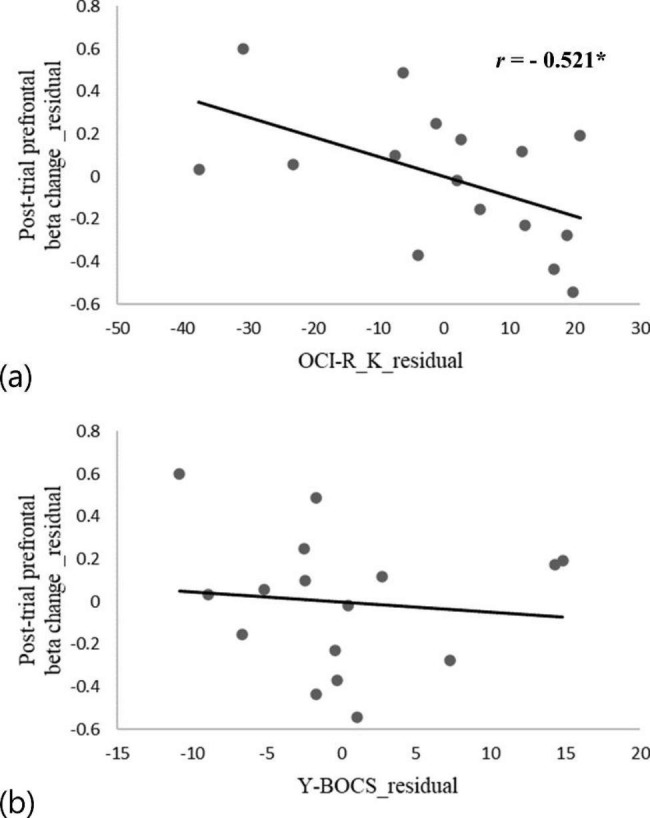
Scatter plots of partial correlations between the post-trial beta power change and obsessive-compulsive symptom severity scores in patients with OCD after controlling for depressive symptoms. The x-axis and y-axis indicate unstandardized residual values from a linear regression analysis of obsessive-compulsive symptom scores and the prefrontal beta power change during the post-trial period after adjusting for MADRS scores, respectively. (**a**) The post-trial prefrontal beta power change and OCI-R-K scores, (**b**) the post-trial prefrontal beta power change and Y-BOCS scores. MADRS, Montgomery-Åsberg Depression Rating Scale; Y-BOCS, Yale-Brown Obsessive Compulsive Scale; OCI-R-K, Korean version of the Obsessive-Compulsive Inventory–Revised. * significant at p < 0.05

In patients with OCD, partial correlation analyses controlling for depressive symptoms were conducted between the obsessive-compulsive symptom severity score and prefrontal beta power change in the post-trial period, which significantly differed according to post hoc ANCOVA. The prefrontal post-trial beta power change was significantly negatively correlated with the OCI-R-K total score in patients with OCD (*r* = -0.521; p = 0.046). No association was found between the prefrontal beta power change and Y-BOCS scores (*r* = -0.108; p = 0.701) [Table 4]. Scatter plots of partial correlations between the post-trial beta power change and each obsessive-compulsive symptom severity score are presented in Fig. 5.

**Table 4:** Partial correlation between the prefrontal post-trial beta power change and obsessive-compulsive symptom severity scores in patients with OCD (n = 16)

Control factor	Symptom severity scores	Beta power change (dB)	
		*r*	p
MADRS	Y-BOCS	− 0.108	0.701
	OCI-R-K	− 0.521	0.046*

OCD, Obsessive-compulsive disorder; MADRS, Montgomery-Åsberg depression rating scale; Y-BOCS, Yale-Brown obsessive compulsive scale; OCI-R-K, Korean version of obsessive-compulsive inventory-revised. * significant at p < 0.05

## Discussion

This study examined changes in prefrontal alpha and beta activity during multiple phases of information processing associated with visuospatial working memory between patients with OCD and HCs using the MEG technique. The main finding of the present study is that the increase in prefrontal beta power during the post-trial period of the working memory task was significantly reduced in patients with OCD compared to HCs. No between-group difference in prefrontal alpha and beta band activity was found in the encoding, retention, and retrieval phases of the working memory task. These results suggest that the post-trial period of the working memory task may be more strongly linked to OCD pathophysiology than the other phases associated with working memory.

To our knowledge, the present MEG study is the first to demonstrate altered neural activity regarding the removal of no-longer-relevant information from working memory in OCD patients. Although removal of information from working memory is proposed to play a crucial role in successful updating of working memory [15], little is known about its roles and neural basis in clinical research. Recently, a “clearing” function of beta band activity has been proposed to halt actions or thoughts during cognitive, perceptual, and motor processes; notably, increased prefrontal beta band activity played a role in maintaining and clearing working memory [21]. An electrophysiological study in monkeys showed increased prefrontal beta bursts at the end of the trial (i.e., after the behavioral response) during a visuospatial working memory task, suggesting that successful inhibitory control through increased prefrontal beta activity may be involved in the removal of no-longer-relevant information from working memory [20]. Based on these observations, the current finding of a significant reduction in the increased prefrontal beta power during the post-trial period in the OCD group suggests failures of inhibitory control to increase beta power when information is no longer needed in OCD patients. In addition, the beta power changes observed during the post-trial period in OCD patients in the present study were inversely correlated with symptom severity assessed by the OCI-R-K, indicating greater reductions in prefrontal beta increases accompanied more severe OCD symptoms. Impairments in prefrontal beta-related inhibitory control during the removal process may be related to the inability to clear unwanted thoughts from working memory and may be a crucial mechanism underlying the symptoms and etiology of OCD.

On the other hand, in the other periods (encoding, retention, and retrieval), no significant differences in prefrontal beta power changes were found between patients with OCD and HCs. Beta activity in the prefrontal cortex has been proposed to play a role in inhibitory control during working memory processes in previous EEG studies, in which beta power was reported to decrease during the encoding phase to enable the encoding of working memory, then slightly increase in the retention phase (compared to the encoding phase) to inhibit irrelevant information from being encoded in working memory, and finally decrease during the retrieval phase to permit the retrieval of information encoded in working memory [20, 21]. Visual inspection of phase-specific increased or reduced patterns of beta band activity (Fig. 3) revealed similar patterns in both the OCD and HC groups in the present study. The findings indicate that OCD patients exhibit impairments of prefrontal beta power (inhibitory control) only in the removal period, not in the encoding, retention, and retrieval phases. In behavioral performance analysis, significantly longer reaction time in patients with OCD, with high accuracy over 90% comparable to HCs, might be associated with compensatory effortful inhibition before the choice response to overcome impaired inhibitory control of the removal process. The assumption of effortful inhibition is supported by the finding that prefrontal alpha power during the retrieval phase was higher in OCD patients than in HCs (p = 0.037), although the significance level did not survive Bonferroni correction (significance threshold set at p < 0.00625).

Regarding prefrontal alpha power, contrary to our expectations, no significant differences were found between patients with OCD and HCs in any of the periods (encoding, retention, retrieval, or post-trial). The limited sample size in the present study may make it difficult to interpret the negative findings regarding alpha power. Alpha oscillations are known to play a role in gating by inhibiting sensory information, inhibiting task-irrelevant information, and protecting working memory from distractors [11, 36, 37]. Although abnormalities in the alpha band in OCD patients have been observed during specific phases of working memory or in resting-state EEG data by several studies [10, 23, 34], findings have been mixed. The inconsistent findings in OCD patients could be attributed to several factors, including task characteristics (e.g., executive demands of the distractor, cognitive load, and complexity), target brain regions, and heterogeneous subject characteristics (e.g., symptom profiles and treatment response) [38]. Regarding task characteristics, the visuospatial DMST in the present study seems to impose few executive demands because it did not involve distractors, and high accuracy was shown in both the OCD and HC groups. An MEG study using the DMST showed reduced frontal alpha modulation during the retention and retrieval phases on the DMST with distractors, while no significant findings regarding alpha power were observed during the task without distractors, in line with our negative results regarding alpha oscillations [23]. Findings of alpha alterations depending on high executive demands, such as the presence of distractors in tasks performed by OCD patients, could suggest that prefrontal alpha alterations in OCD patients may be involved in executive inhibition of task-irrelevant distractions during memory maintenance rather than the removal of no-longer-needed information. Regarding brain region, the present study targeted only the prefrontal cortex. As the inhibitory function of alpha oscillations is responsible for the active inhibition of sensory regions, including the occipital area, during working memory maintenance, the brain regions encompassing sensory cortex and their connectivity should be considered [11, 36]. In addition, regarding subject characteristics, heterogeneous phenotypic and biological characteristics among individuals with OCD such as symptom dimensions, clinical course, comorbidities, and underlying neural substrates might lead to inconsistent results. The present patients with OCD who were recruited from a tertiary hospital (referred from a primary hospital) had relatively severe symptoms and had one or more symptom dimensions. Stratifying patients with OCD into more homogeneous subtypes was not applied to the current study due to the small sample size. Further research using working memory paradigms and manipulating cognitive load in a larger homogeneous sample is needed to more clearly determine if there are phase-specific alterations of neural oscillations in OCD patients during working memory processes.

Taken together, these findings suggest impaired prefrontal beta-related inhibitory control of the removal of no-longer-relevant information from working memory in patients with OCD. It may explain why people suffering from unwanted intrusive thoughts are unable to clear their obsessions from working memory. A recent multilevel meta-analysis proposed a model of memory deficits in OCD, with maintenance and updating (top-down) and perceptual integration (bottom-up) predicting the memory performance of OCD patients [39]. The present findings of impaired inhibitory control (through prefrontal beta power) of no-longer-relevant information in OCD patients support the top-down framework of the model. Further research is needed to confirm the role of task-phase-specific beta oscillations in OCD and elucidate how inhibitory control related to the information removal process contributes to symptom manifestations and etiology of OCD.

There are several limitations of this study. First, the sample size was relatively small. In this study, the estimated effect size (using G*Power version 3.1.9.7 [40], with the following parameters: 36 participants, a power of 0.8, and 4 response variables) was f^2^(V) = 0.39. Although the assumption of the effect size was consistent with the results of previous MEG and EEG studies in which the effect sizes f^2^ generally ranged from 0.31 to 0.48 [34, 41, 42], the sample size may not be enough to detect differences in some variables with a small effect size. Therefore, the results of the present study need to be replicated with a study with a larger sample size. Second, we could not entirely exclude the possible effects of psychotropic medication on neural oscillations. Most patients in the present study were taking selective serotonin reuptake inhibitors, and nearly half of them were also taking benzodiazepines. Alterations in neural oscillations induced by psychotropic medication are reportedly complex and depend on the drug class, individual drugs, and duration of treatment [43–46]. Although the between-group difference in the post-trial prefrontal beta power change remained significant with a large effect size when the use of benzodiazepines was included in the analysis as a covariate (F_1_ = 6.961; p = 0.013; η_p_^2^ = 0.179), the present findings need to be confirmed in further studies of drug-naïve patients. Third, our analyses were based on the sensor level; therefore, the specific brain regions with differences could not be identified. Further source analysis can provide brain region-based information about the pathophysiology of OCD. Fourth, anticipation of feedback might have affected brain activity during the post-trial period. However, brain electrical activity related to anticipation of feedback represented by the prefeedback stimulus preceding negativity (SPN) was not high in the prefrontal area [47]. Since both groups exhibited high accuracy (more than 90%) in the task used in the present study and only correct trials were included in the analysis, the present results are unlikely to be affected by feedback. Finally, since we included only young men to reduce heterogeneity among participants, these results might not be applicable to women or other age groups.

## Conclusions

In this study, the beta power increase during the post-trial period of the DMST in patients with OCD was reduced compared to that in HCs in the prefrontal region. In addition, the greater reduction in the prefrontal beta power increase in patients with OCD was related to more severe OCD symptoms. As beta oscillations occurring at high levels at the end of a trial during working memory tasks are implicated in the removal of information from working memory, the present findings suggest that OCD patients may exhibit impairments in prefrontal beta-related inhibition of the removal of no-longer-relevant information. This could explain why OCD patients suffering from intrusive thoughts fail to expel their obsessions. Additional studies are required to explore how impairments in the removal of information from working memory are associated with the pathophysiology of OCD.

## Data Availability

Raw data are not publicly shared because the participants of this study did not give written consent for their data to be shared openly. However, the datasets analyzed during the current study are available from the corresponding authors upon reasonable request.

## References

[1] American Psychiatric Association (2013). Diagnostic and statistical manual of mental disorders.

[2] Chamberlain SR, Blackwell AD, Fineberg NA, Robbins TW, Sahakian BJ (2005). The neuropsychology of obsessive compulsive disorder: the importance of failures in cognitive and behavioural inhibition as candidate endophenotypic markers. Neurosci Biobehavioral Reviews.

[3] Maia TV, Cooney RE, Peterson BS (2008). The neural bases of obsessive-compulsive disorder in children and adults. Dev Psychopathol.

[4] Posner J, Marsh R, Maia TV, Peterson BS, Gruber A, Simpson HB (2014). Reduced functional connectivity within the limbic cortico-striato-thalamo-cortical loop in unmedicated adults with obsessive-compulsive disorder. Hum Brain Mapp.

[5] Robbins TW, Vaghi MM, Banca P (2019). Obsessive-compulsive disorder: puzzles and prospects. Neuron.

[6] Snyder HR, Kaiser RH, Warren SL, Heller W (2014). Obsessive-compulsive disorder is Associated with Broad impairments in executive function: a Meta-analysis. Clin Psychol Sci.

[7] Abramovitch A. 149Neuropsychological Function in OCD. In: *Obsessive-compulsive Disorder: Phenomenology, Pathophysiology, and Treatment* edn. Edited by Pittenger C, Pittenger C: Oxford University Press; 2017: 0.

[8] Abramovitch A, Abramowitz JS, Mittelman A (2013). The neuropsychology of adult obsessive–compulsive disorder: a meta-analysis. Clin Psychol Rev.

[9] Shin NY, Lee TY, Kim E, Kwon JS (2014). Cognitive functioning in obsessive-compulsive disorder: a meta-analysis. Psychol Med.

[10] Perera MPN, Bailey NW, Herring SE, Fitzgerald PB (2019). Electrophysiology of obsessive compulsive disorder: a systematic review of the electroencephalographic literature. J Anxiety Disord.

[11] Miller EK, Lundqvist M, Bastos AM (2018). Working Memory 2.0. Neuron.

[12] Chai WJ, Abd Hamid AI, Abdullah JM. Working Memory from the psychological and Neurosciences Perspectives: a review. Front Psychol 2018, 9. 10.3389/fpsyg.2018.00401 PMC5881171 29636715

[13] Anderson MC, Hanslmayr S (2014). Neural mechanisms of motivated forgetting. Trends Cogn Sci.

[14] Kim H, Smolker HR, Smith LL, Banich MT, Lewis-Peacock JA (2020). Changes to information in working memory depend on distinct removal operations. Nat Commun.

[15] Lewis-Peacock JA, Kessler Y, Oberauer K (2018). The removal of information from working memory. Ann N Y Acad Sci.

[16] Taylor R, Tomić I, Aagten-Murphy D, Bays PM. Working memory is updated by reallocation of resources from obsolete to new items. Attention, Perception, & Psychophysics; 2022. 10.3758/s13414-022-02584-2 PMC7614821 36253588

[17] Gross J (2019). Magnetoencephalography in Cognitive Neuroscience: a primer. Neuron.

[18] Daniel TA, Katz JS, Robinson JL (2016). Delayed match-to-sample in working memory: a BrainMap meta-analysis. Biol Psychol.

[19] Supek S, Aine CJ. Magnetoencephalography: from signals to dynamic cortical networks. Springer Berlin Heidelberg; 2014.

[20] Lundqvist M, Herman P, Warden MR, Brincat SL, Miller EK (2018). Gamma and beta bursts during working memory readout suggest roles in its volitional control. Nat Commun.

[21] Schmidt R, Herrojo Ruiz M, Kilavik BE, Lundqvist M, Starr PA, Aron AR (2019). Beta oscillations in Working Memory, Executive Control of Movement and Thought, and sensorimotor function. J Neurosci.

[22] Ciesielski KT, Hamalainen MS, Lesnik PG, Geller DA, Ahlfors SP (2005). Increased MEG activation in OCD reflects a compensatory mechanism specific to the phase of a visual working memory task. NeuroImage.

[23] Ciesielski KT, Hämäläinen MS, Geller DA, Wilhelm S, Goldsmith TE, Ahlfors SP (2007). Dissociation between MEG alpha modulation and performance accuracy on visual working memory task in obsessive compulsive disorder. Hum Brain Mapp.

[24] Choe AY, Soon Taeg H, Ji Hae K, Kwang Bai P, Jean Yung C, Sang Hwang H (2014). Validity of the K-WAIS-IV short forms. Korean J Clin Psychol.

[25] Wechsler DK, Hwang SK, Kim JK, Park KK, Choi JK, Hong SK (2012). (K-WAIS®-IV) Korean-Wechsler Adult Intelligence Scale-IV.

[26] First MB, Spitzer R, L GM, Williams JBW (1996). Structured clinical interview for DSM-IV Axis I Disorders, Clinical Version (SCID-CV).

[27] Montgomery SA, Asberg M (1979). A new depression scale designed to be sensitive to change. Br J Psychiatry.

[28] Ahn YM, Lee KY, Yi JS, Kang MH, Kim DH, Kim JL, Shin JH, Shin HK, Yeon BK, Lee JH et al. A validation study of the korean-version of the Montgomery-Asberg Depression Rating Scale. J Korean Neuropsychiatr Assoc 2005, 44(4).

[29] Goodman WK, Price LH, Rasmussen SA, Mazure C, Delgado P, Heninger GR, Charney DS (1989). The Yale-Brown Obsessive compulsive scale. II. Validity. Arch Gen Psychiatry.

[30] Bloch MH, Landeros-Weisenberger A, Rosario MC, Pittenger C, Leckman JF (2008). Meta-analysis of the symptom structure of obsessive-compulsive disorder. Am J Psychiatry.

[31] Lim JS, Kim SJ, Jeon WT, Cha KR, Park JH, Kim CH (2008). Reliability and validity of the korean version of obsessive-compulsive inventory-revised in a non-clinical sample. Yonsei Med J.

[32] Konorski J (1959). A new method of physiological investigation of recent memory in animals. Bull Acad Pol Sci.

[33] Delorme A, Makeig S (2004). EEGLAB: an open source toolbox for analysis of single-trial EEG dynamics including independent component analysis. J Neurosci Methods.

[34] Park JY, Lee J, Park HJ, Kim JJ, Namkoong K, Kim SJ (2012). Alpha amplitude and phase locking in obsessive-compulsive disorder during working memory. Int J Psychophysiol.

[35] Cohen J (1988). Statistical power analysis for the behavioral Sciences.

[36] Jensen O, Mazaheri A (2010). Shaping functional architecture by oscillatory alpha activity: gating by inhibition. Front Hum Neurosci.

[37] Roux F, Uhlhaas PJ (2014). Working memory and neural oscillations: α-γ versus θ-γ codes for distinct WM information?. Trends Cogn Sci.

[38] Harkin B, Kessler K (2011). The role of working memory in compulsive checking and OCD: a systematic classification of 58 experimental findings. Clin Psychol Rev.

[39] Harkin B, Persson S, Yates A, Jauregi A, Kessler K (2023). Top-down and bottom-up contributions to memory performance in OCD: a multilevel meta-analysis with clinical implications. J Psychopathol Clin Sci.

[40] Faul F, Erdfelder E, Buchner A, Lang AG (2009). Statistical power analyses using G*Power 3.1: tests for correlation and regression analyses. Behav Res Methods.

[41] Thoma RJ, Hanlon FM, Moses SN, Edgar JC, Huang M, Weisend MP, Irwin J, Sherwood A, Paulson K, Bustillo J (2003). Lateralization of auditory sensory gating and neuropsychological dysfunction in schizophrenia. Am J Psychiatry.

[42] Oda Y, Onitsuka T, Tsuchimoto R, Hirano S, Oribe N, Ueno T, Hirano Y, Nakamura I, Miura T, Kanba S (2012). Gamma band neural synchronization deficits for auditory steady state responses in bipolar disorder patients. PLoS ONE.

[43] Knott V, Mahoney C, Kennedy S, Evans K (2002). EEG correlates of acute and chronic paroxetine treatment in depression. J Affect Disord.

[44] Nissen TD, Laursen B, Viardot G, l’Hostis P, Danjou P, Sluth LB, Gram M, Bastlund JF, Christensen SR, Drewes AM (2020). Effects of Vortioxetine and Escitalopram on Electroencephalographic Recordings - a randomized, crossover trial in healthy males. Neuroscience.

[45] Tarn M, Edwards JG, Sedgwick EM (1993). Fluoxetine, amitriptyline and the electroencephalogram. J Affect Disord.

[46] Yamadera H, Kato M, Ueno T, Tsukahara Y, Okuma T (1993). Pharmaco-EEG mapping of diazepam effects using different references and absolute and relative power. Pharmacopsychiatry.

[47] Brunia CH, Hackley SA, van Boxtel GJ, Kotani Y, Ohgami Y (2011). Waiting to perceive: reward or punishment?. Clin Neurophysiol.

